# Evaluation of the EMPAR study population on the basis of metabolic phenotypes of selected pharmacogenes

**DOI:** 10.1038/s41397-022-00268-6

**Published:** 2022-01-31

**Authors:** Jochen Fracowiak, Tatjana Huebner, Steffen Heß, Christoph Roethlein, Daria Langner, Udo Schneider, Felix Falkenberg, Catharina Scholl, Roland Linder, Julia Stingl, Britta Haenisch, Michael Steffens

**Affiliations:** 1 Research Division, Federal Institute for Drugs and Medical Devices, Bonn, North Rhine-Westphalia Germany; 2 German Center for Neurodegenerative Diseases (DZNE), Bonn, North Rhine-Westphalia Germany; 3 Techniker Krankenkasse (TK), Hamburg, Germany; 4 Institute for Clinical Pharmacology, RWTH Aachen University, Aachen, North Rhine-Westphalia Germany; 5 Center for Translational Medicine, Medical Faculty, University of Bonn, Bonn, North Rhine-Westphalia Germany

**Keywords:** Predictive markers, Genetic markers

## Abstract

The impact of genetic variability of pharmacogenes as a possible risk factor for adverse drug reactions is elucidated in the EMPAR (Einfluss metabolischer Profile auf die Arzneimitteltherapiesicherheit in der Routineversorgung/English: influence of metabolic profiles on the safety of drug therapy in routine care) study. EMPAR evaluates possible associations of pharmacogenetically predicted metabolic profiles relevant for the metabolism of frequently prescribed cardiovascular drugs. Based on a German study population of 10,748 participants providing access to healthcare claims data and DNA samples for pharmacogenetic assessment, first analyses were performed and evaluated. The aim of this first evaluation was the characterization of the study population with regard to general parameters such as age, gender, comorbidity, and polypharmacy at baseline (baseline year) as well as important combinations of cardiovascular drugs with relevant genetic variants and predicted metabolic phenotypes. The study was registered in the German Clinical Trials Register (DRKS) on July 6, 2018 (DRKS00013909).

## Introduction

Preemptive pharmacogenetic testing in routine care can identify risk factors for adverse drug reactions (ADRs) related to genetic variability and thus decrease the risk of ADRs that cause high costs and jeopardize the health of patients. About 5–15% of hospitalizations are linked to ADRs [1, 2]. However, the implementation of preemptive PGx testing for preventive measures in clinical laboratories faces many barriers, especially the lack of data from studies with large study populations, the complexity of PGx analysis and reimbursement policies [3].

Routine care records provide useful data on the utilization of health services and health expenditure. For this cooperation project, such relevant routine care records are provided after the successful recruitment of Techniker Krankenkasse (TK) insurants by the TK, a large statutory health insurance company with about 10.8 million insurants in Germany [4]. TK insurants recruited, furthermore, provided genetic samples and permission for assessment of their genetic data on pharmacogenes.

One major group of pharmacogenes with genetic variability affecting drug response of several lead substances is the Cytochrome P450 protein family that is responsible for about 75% of all phase I drug reactions in drug metabolism [5]. Thus, cytochromes such as CYP2C9, CYP2C19, CYP3A4, and CYP3A5 are considered in the study evaluations. Furthermore, genetic variants of the vitamin K epoxide reductase complex subunit 1 (*VKORC1*) involve a risk for serious ADRs such as bleeding events in drug therapy with phenprocoumon and warfarin [6] and variants of transporters like Solute Carrier Organic Anion Transporter Family Member 1B1 (*SLCO1B1*) and ATP Binding Cassette Subfamily B Member 1 (*ABCB1*), which affect the transport of statins are of interest [7–9]. Several databases are in place to support pharmacogenetic assessments, such as the Single Nucleotide Polymorphism Database (dbSNP) for collecting and archiving data on genome variations and frequencies from large genome studies [10]. Research consortia such as PharmVar [11] and databases such as The Pharmacogenomics Knowledge Base (PharmGKB) [12] collect, review, and provide valid information on known variations of pharmacogenes, which can be further used to deduce their metabolic phenotype. Furthermore, PharmGKB provides information on gene–drug relationships that are clinically actionable and therefore potentially require variant-specific clinical action [13]. In this evaluation, pharmacogenetic variants of the high PharmGKB Clinical Annotation Levels of Evidence 1A or 1B, therefore, are referred to as actionable variants [14]. Here, we present the first data on pharmacogenetic analyses in the EMPAR (Einfluss metabolischer Profile auf die Arzneimitteltherapiesicherheit in der Routineversorgung/English: influence of metabolic profiles on the safety of drug therapy in routine care) study involving the so-far largest collection of pharmacogenetic profiles with health insurance data in Germany. In this first evaluation, a focus is set on variants of genes with an impact on absorption, distribution, metabolism, and elimination of lead substances relevant for cardiovascular conditions. Cardiovascular diseases are events of elevated prevalence among elderly people in Germany and one of the leading causes of mortality in developed countries [5, 6]. Therefore, analyses were focused on the anticoagulant/antiplatelet and the cholesterol-lowering drug collective of the EMPAR study population. Furthermore, routine care records of TK insurants such as age, gender, medication, and diagnoses in the year prior to prescription of the main medication of study interest were analyzed.

## Methods

### Study design and recruitment

EMPAR recruitment was initiated in 2018 in Germany with a target sample size of about 10,000 adult study participants insured by TK. The present study population comprises three patient collectives. The two cardiovascular drug collectives (anticoagulant/antiplatelet and cholesterol-lowering drug collective) were of major interest for essential evaluations in this study. The International Classification of Diseases (ICD-10) Y57.9! collective serves for screening for new relevant single-nucleotide polymorphisms (SNPs) associated with ADRs in future investigations and was only addressed in this first evaluation with regard to general characteristics such as age, gender, polypharmacy, morbidity, and actionable variants. Further details on the study design were published by Huebner et al. and in the German Clinical Trials Register [15, 16]. For an additional recruitment phase in 2020, the period of initial prescription for the anticoagulant/antiplatelet and cholesterol-lowering drug collective was extended until the end of 2017 in order to reach the target sample size. Recruited participants provided written informed consent and sent a questionnaire and buccal swab samples to the Deutsche Knochenmarkspenderdatei Life Science Lab GmbH (Dresden, Germany) for sample collection and DNA extraction. DNA samples were genotyped via the pharmacogenomics services of Agena Bioscience Inc. (Hamburg, Germany) for the application of the iPLEX^®^ PGx 74 and the VeriDose^®^ CYP2D6 CNV panel [17].

### Data quality control

Genotype data of 67 markers, covering 19 genes [18], were quality controlled by computing marker call rates and minor allele frequencies (MAF). The latter were compared with data of the Caucasian population obtained from dbSNP. Allele frequencies were calculated by PLINK version 1.9 and tested for Hardy–Weinberg equilibrium (HWE) [19]. Due to data policy reasons, rs7412 and rs429358 (*APOE*) were excluded from analyses.

### Evaluations of collective characteristics

Investigated parameters such as gender, age, diagnoses based on ICD-10 system, and medication were obtained from anonymized TK healthcare claims data. A time window of 1 year before individual prescription of the main medication was set for evaluations of population characteristics at baseline of the anticoagulant/antiplatelet and cholesterol-lowering drug collective. The Y57.9!-diagnosis collective was investigated up to 1 year before the first appearance of a Y57.9 or Y57.9!-diagnosis entry.

For an initial study population and collective description, ICD-10 entries of the individual baseline year were taken into account. Long-term polypharmacy was defined by a prescription frequency of ≥5 medications, observed in a consecutive sequence of four quarters (baseline year). For comorbidity and polypharmacy assessment, stratified pharmacoepidemiological analyses of the three study collectives (cholesterol reducer, anticoagulant/antiplatelet, and Y57.9! diagnosis) were performed by age (young: <36; moderate: 36–60; old: >60) and gender (male, female). Comorbidity was calculated according to Elixhauser, implemented in the R-package comorbidity (version 0.5.3) [20]. All statistics for collective characterization were carried out using R 3.6.3 and Python 3.

### Evaluations of pharmacogenetic data

PGx markers of study interest (Supplement 1 – Table 1) were used for further evaluations with regard to pharmacogenetic data and associated main medication of study participants. Pharmacogenes covered by single markers only (*VKORC1, SLCO1B1*) were directly translated into their corresponding star allele nomenclature. Pharmacogenes covered by more than one marker were firstly computational phased to haplotypes by applying PLINK’s EM-algorithm and subsequently combined to diplotypes. The resulting diplotypes were translated into the star allele nomenclature, according to PharmGKB and scientific literature [12, 19]. Corresponding metabolic phenotypes were determined based on the PhamGKB Diplotype-Phenotype translation tables. In case of *CYP2D6*, diplotypes were computed by additionally considering the gene copy number data and non-functional hybrid allele information (*36, *13, and *68) (Agena Bioscience VeriDose^®^ *CYP2D6* CNV Panel). Phenotypes were derived by using the activity scoring system, according to the CYP2D6 consensus definition, published by Caudle et al. [21]. All computed phenotypes primarily relevant for the cardiovascular drug metabolism or transport were compared with the corresponding marker frequencies in dbSNP and to frequencies reported in similar studies.

In this first assessment, the EMPAR study population was analyzed for allele distributions of actionable variants (variants of PharmGKB evidence level 1A or 1B [14]) relevant for the response to therapy with anticoagulants, antiplatelets or cholesterol reducers of study interest (main medication). This criterion was met by 13 markers (Supplement 2, Actionable variants). The identified actionable markers and the according cardiovascular indications were applied for filtering and assessment of allele distributions.

For further analyses of the cardiovascular drug collectives, also variants with lower evidence levels were considered (Supplement 1 – Table 1). For the anticoagulant/antiplatelet and cholesterol-lowering drug collective, respective pharmacogenes that could interact and thus may increase the impact on drug response were analyzed pairwise with regard to the occurrence and frequencies of diplotype combinations. Furthermore, diplotypes of six combinations of *CYP3A4*, *CYP3A5*, *SLCO1B1*, and *ABCB1* were analyzed. In phenotype analyses, frequencies of extreme phenotypes (ultrarapid or poor function) in combination with the affected prescribed main medication were assessed for each collective. All statistics for the evaluations of pharmacogenetic data were carried out by using Python 3.

## Results

### Recruitment

**Fig. 1:**
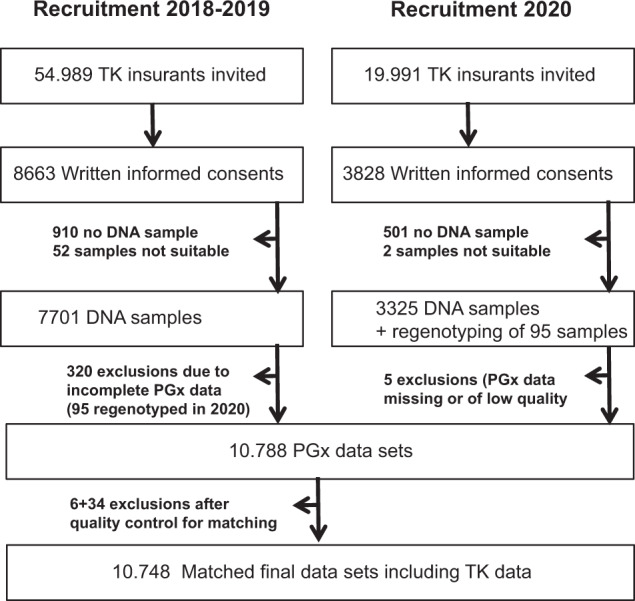
Recruitment scheme of the EMPAR study in 2018–2020. Dropouts occurred in the provision of suitable DNA samples (11.1% in 2018–2019 and 13.1% in 2020), due to insufficient quality or a lack of genotype data (2.2%) and due to insufficient quality of healthcare claims data for matching (0.4%). PGx pharmacogenetic.

In total, 54,989 TK insurants were invited to participate in 2018. In 2020, the TK invited further 19,991 insurants with an initial prescription of anticoagulants/antiplatelets and cholesterol reducers in 2016–2017 to participate. About 16.7% of the eligible TK insurants consented to be included in the EMPAR study. About 11,026 (88.27%) of those who sent in their written informed consent provided a DNA sample for pharmacogenetic genotyping. In total, data on relevant pharmacogenetic SNPs and copy number variations could be obtained from 10,791 participants. However, genotype data of 10,788 participants were suitable for further analyses according to data quality parameters (Fig. 1).

### Data quality control

**Fig. 2:**
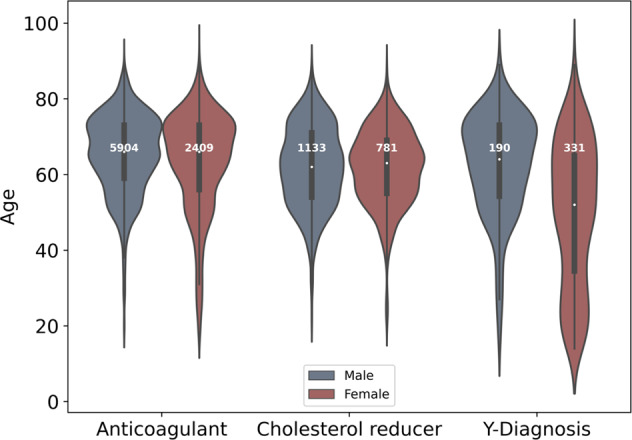
Age and sex evaluation. Age distributions of male and female participants in each study collective of the complete study population (anticoagulant: anticoagulant/antiplatelet collective, cholesterol reducer: cholesterol-lowering drug collective, Y-diagnosis: Y57.9! diagnosis collective) at baseline.

**Fig. 3:**
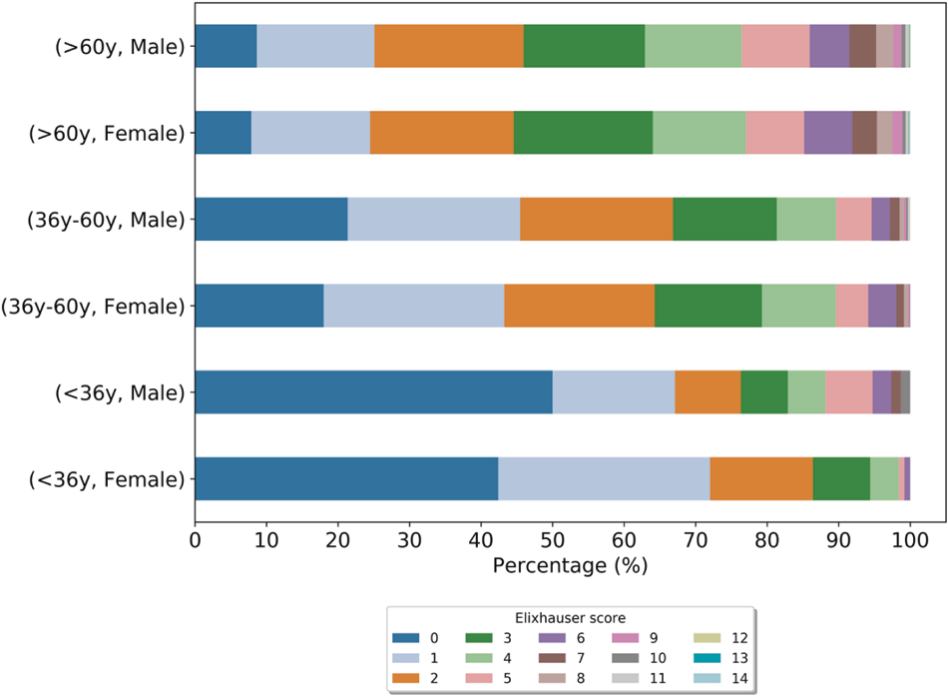
Elixhauser comorbidity score distributions (in %). All diagnosis codes (ICD-10) of the individual baseline year were taken into account. The score distribution reflects the comorbidity status of the complete study population in the baseline year grouped by age and gender.

An overall marker call rate of 98.4% was calculated (Supplement 1 – Fig. 1). Moreover, 49 markers were tested to be in HWE and 18 significantly deviated from HWE (Supplement 1 – Fig. 2). The calculated MAFs in the EMPAR population showed no conspicuous deviation from the MAFs of the respective markers reported in dbSNP for the Caucasian European (CEU) population (Supplement 1 – Fig. 3). Three participants were dropped from the study due to low marker call rates.

### Study population characteristics

Genotype data available from 10,788 participants were merged with the according healthcare claims data. Thereby, data records of six participants did not meet the necessary quality criteria in terms of ICD-10 code entry Y57.9! for further analyses. These participants were excluded from the study. Furthermore, subsequently, 34 participants of the cholesterol-lowering drug collective with prescriptions of combination products that include statins in the baseline year were excluded due to the according exclusion criterion for this collective (13.9% overall dropout). In sum, healthcare claims data and phenotype data of 10,748 participants were successfully merged (Fig. 1). The study population comprises 8313 (77.3%) participants of the anticoagulant/antiplatelet collective, 1914 (17.8%) participants of the cholesterol-lowering drug collective and 521 (4.8%) participants of the Y57.9!-diagnosis collective (Fig. 2).

In the evaluation of general collective characteristics, a stratified analysis of basal cohort morbidity by age (young: <36; moderate: 36–60; old: >60) and gender among all collectives (Supplement 3) elucidated an increasing number of diagnoses with age. This general tendency seems to be supported by the comorbidity distributions (Fig. 3) representing the mortality prediction in terms of the Elixhauser score that was used to assess the health situation before prescription of the main medication. Clear tendencies of higher risk scores from young to old participants were observed with marginal differences between males and females. Among the three collectives, the median ages of male and female participants in the baseline year were all above 60 years (64.73 years on average (σ = 11.78)), except in the Y57.9!-diagnosis collective with a median age of 52 years for female participants.

The comorbidity analysis of the complete study population revealed 10,227 (93.9%) participants with outpatient and inpatient ICD-10 entry in the individual baseline year. A total of 6226 different outpatient and inpatient ICD-10 diagnoses were observed. Overall, essential (primary) hypertension (I10.90), which is an important risk factor for different cardiovascular conditions [22], was the most frequent diagnosis in the mid and old age groups of all cohorts of the study population. Further frequently detected diagnoses relevant for cardiovascular conditions were disorders of lipoprotein metabolism and other lipidemias (e.g., E78.0, E78.5), benign essential hypertension (I10.00) and diabetes mellitus, type 2 (E11.90) (Supplement 3).

**Fig. 4:**
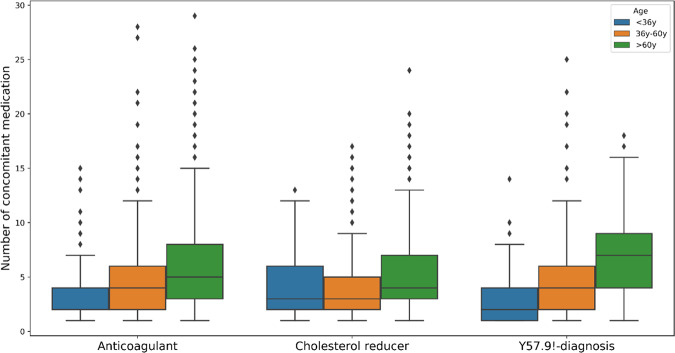
Abundance of concomitant medication prescribed during the individual baseline year grouped by age and collective. Anticoagulant: anticoagulant/antiplatelet.

An investigation in the period of 1 year prior to prescription of the main medication (anticoagulant collective and cholesterol-lowering drug collective) or prior to Y57.9!-diagnosis (Y57.9!-diagnosis collective) on the number of concomitant medication prescribed in the same quarter revealed that 9746 (90.7%) participants obtained at least one prescription in the individual baseline year and 3635 (33.8%) were identified with ≥5 prescriptions in at least one quarter of the baseline year. A stratified assessment between concomitant medication, age groups, and collectives illustrates the highest median prescription number for the old age groups (>60 years). Thereby, the highest counts of concomitant prescriptions in the baseline year were identified for the Y57.9!-diagnosis collective (Median: 7 prescriptions). In the cholesterol reducer collective, a median of 4 prescriptions and in the anticoagulant collective, a median of 5 prescriptions was identified in the old age group (Fig. 4). On average, 6.9 (σ = 5.53) different medications were counted per person. An analysis of long-term polypharmacy in the EMPAR population showed that 21% of participants had prescriptions of at least two medications within each quarter over the baseline year and about 242 (2.3% of study participants) were affected by long-term polypharmacy at baseline (Table 1).

**Table 1:** Participants with prescribed medication in a consecutive sequence of four quarters at baseline (baseline year).

Number of medication	Number of participants	%
1	2155	20.05
2	1166	10.85
3	571	5.31
4	279	2.60
5	135	1.26
6	67	0.62
7	23	0.21
8	10	0.09
9	6	0.06
10	1	0.01

In the study population, a total of 113,203 prescriptions were counted within the baseline year, thereby also prescriptions of antagonists for CYP2C9 (11.03%), CYP2C19 (8.79%), VKORC1 (0.21%), SLCO1B1 (21.92%), ABCB1 (31.35%), CYP3A4 (13.33%), and CYP3A5 (0.04%) were identified. In addition, a high percentage of participants within the cardiovascular drug collectives was identified with relevant prescriptions of inhibitors and antagonists that have a functional effect on the cytochromes, catalytic subunits, and transporters under study (Table 2). Especially, for clopidrogrel, simvastatin, and phenprocoumon, clinical annotations of a high level of evidence with regard to drug–gene pairs such as clopidrogrel-*CYP2C19*, simvastatin-*SLCO1B1* and phenprocoumon-*VKORC1* are present (Supplement 2, Medication). Clopidrogrel, simvastatin, and phenprocoumon were identified among the most frequently prescribed main medication in the EMPAR study population (Supplement 1 – Fig. 4). Therefore, they were of special interest concerning pharmacogenetic assessments and may also provide a sufficient sample size for future pharmacoeconomic assessments.

**Table 2:** Number and percentage of participants within the study population and study collectives with prescriptions of medication with functional impact on the cytochromes, catalytic subunit, and transporters under study within the baseline year.

Collective	Gene	Participants with inhibitor or antagonist prescription	Percentage within collective
Complete study population	*CYP2C9*	4058	37.76
	*CYP2C19*	3677	34.21
	*VKORC1*	110	1.02
	*SLCO1B1*	6327	58.87
	*ABCB1*	7589	70.61
	*CYP3A4*	5416	50.39
	*CYP3A5*	35	0.33
Anticoagulant/antiplatelet	*CYP2C9*	3437	41.35
	*CYP2C19*	2913	35.04
	*VKORC1*	0	0.00
	*SLCO1B1*	5071	61.00
	*ABCB1*	5999	72.16
	*CYP3A4*	4232	50.91
	*CYP3A5*	23	0.28
Cholesterol-lowering drug	*CYP2C9*	418	21.84
	*CYP2C19*	542	28.32
	*VKORC1*	74	3.87
	*SLCO1B1*	931	48.64
	*ABCB1*	1222	63.85
	*CYP3A4*	893	46.66
	*CYP3A5*	9	0.47
Y57.9!	*CYP2C9*	203	38.96
	*CYP2C19*	222	42.61
	*VKORC1*	36	6.91
	*SLCO1B1*	325	62.38
	*ABCB1*	368	70.63
	*CYP3A4*	291	55.85
	*CYP3A5*	3	0.58

Antagonists and inhibitors were identified according to respective entries on DrugBank Online, a database for drug and drug target information.

### Pharmacogenetic characteristics

**Fig. 5:**
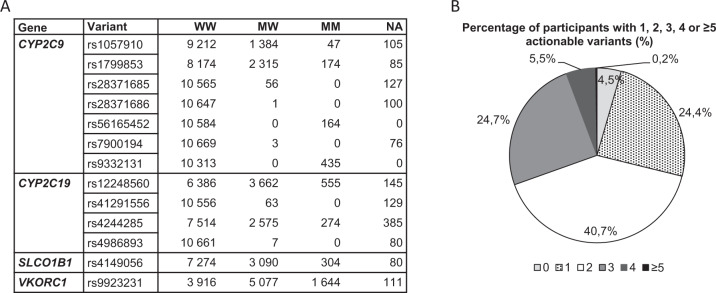
Relevant actionable variants in the EMPAR population. **A** Number of carriers of pharmacogenetic markers with PharmGKB Evidence level 1A/1B with regard to cardiovascular indications. Major allele here defined as wild type (W); M: minor allele or actionable variant respectively; NA: information not available; MW: actionable variant/wild type. **B** Proportions of participants in the complete study population with 1, 2, 3, 4, and ≥5 actionable variants shown in **A**.

The analysis of the actionable variants reflected that 95.5% of the participants of the EMPAR study population were carrier of at least one actionable marker (Fig. 5). Seven of the 13 analyzed markers were monomorphic and corresponded to the wild type (Fig. 5A). Pharmacogenes primarily relevant (Supplement 1 – Table 1) with regard to the considered cardiovascular drugs were analyzed on diplotype (star nomenclature) and metabolic phenotype level. All metabolic phenotype distributions were comparable to reference (PharmGKB) or other study data in terms of the metabolic profile frequencies (Supplement 1 – Figs. 5–7). However, differences in metabolic profile distributions were detected as expected, especially for CYP2D6 as a result of the changed phenotype-scoring algorithm according to the inclusion of VeriDose^®^ CYP2D6 CNV panel data.

Pairwise analysis of diplotypes of *CYP2C19*, *CYP2C9*, *ABCB1*, *VKORC1* concerning the combination frequencies in the anticoagulant collective (Supplement 1 – Figs. 8 and 9) showed that relevant homozygous combinations that deviate from the normal function were rarely present.

Similar results could be observed in the cholesterol reducer collective for a pairwise analysis of selected combinations of *CYP3A4*, *CYP3A5*, *SLCO1B1*, and *ABCB1* (Supplement 1 – Fig. 10). Further collective analysis was performed considering extreme phenotypes (ultrarapid or poor function) (Table 3). Thereby, the focus in this first survey was on drug–gene combinations with sufficient evidence of impact according to PharmGKB clinical annotations (1A-3, Supplement 2, Medication) [23]. In the calculation for the anticoagulant/antiplatelet collective, 41.6% of participants of this subgroup were identified with at least one extreme phenotype of CYP2C9, CYP2C19, VKORC1, or ABCB1 in combination with prescriptions of anticoagulants or antiplatelet medication. In the large anticoagulant/antiplatelet collective clinically important drug–gene pair fractions were identified for clopidogrel or clopidogrel/acetylsalicylic acid + CYP2C19 poor metabolizer (PM)/ultrarapid metabolizer (UM) (2.1%) with evidence level 1A and phenprocoumon + *VKORC1* rs9923231 TT (2.6% with poor function) with The Clinical Pharmacogenetics Implementation Consortium (CPIC^®^) evidence level 1B (Supplement 2). An analog approach was applied for all participants of the cholesterol reducer collective. Thereby, 1699 of 1914 participants (88.8%) were identified with an extreme phenotype of *CYP3A4, CYP3A5, SLCO1B1* or in case of *ABCB1* being homozygous alternating (rs1045642_GG). Only 0.8% of participants of the cholesterol-lowering drug collective were identified with the drug–gene combination simvastatin + SLCO1B1 PM (Table 3), the highest evidence-based drug–gene combination according to CPIC (1A). The drug–gene combination simvastatin + rs1045642_GG (*ABCB1*) with CPIC evidence level 2A occurred in 10.1% of the analyzed participants of the cholesterol-lowering drug collective. Further analyzed drug–gene combinations (Table 3) mentioned by CPIC, but with lower levels of evidence, e.g., related to CYP3A4/5, are listed in Supplement 2.

**Table 3:** Proportion of participants with relevant prescriptions (main medication) and extreme phenotypes of the evaluated cytochromes, catalytic subunit, and transporters in the cardiovascular drug collectives.

Collective	Prescription	Extreme phenotype (predicted)	Gene	Participants affected	Percentage (within collective)
A/T	Apixaban	PF	*ABCB1*	229	2.8
	Apixaban	PF	*VKORC1*	143	1.7
	Apixaban	PM	*CYP2C9*	38	0.5
	Apixaban	PM	*CYP2C19*	23	0.3
	Apixaban	UM	*CYP2C19*	59	0.7
	Clopidogrel	PF	*ABCB1*	523	6.3
	Clopidogrel	PF	*VKORC1*	333	4.0
	Clopidogrel	PM	*CYP2C9*	96	1.2
	Clopidogrel	PM	*CYP2C19*	56	0.7
	Clopidogrel	UM	*CYP2C19*	113	1.4
	Clopidogrel+ASS	PF	*ABCB1*	10	0.1
	Clopidogrel+ASS	PF	*VKORC1*	10	0.1
	Clopidogrel+ASS	PM	*CYP2C9*	2	0.0
	Clopidogrel+ASS	PM	*CYP2C19*	1	0.0
	Clopidogrel+ASS	UM	*CYP2C19*	5	0.1
	Dabigatranetexilat	PF	*ABCB1*	63	0.8
	Dabigatranetexilat	PF	*VKORC1*	37	0.4
	Dabigatranetexilat	PM	*CYP2C19*	7	0.1
	Dabigatranetexilat	PM	*CYP2C9*	7	0.1
	Dabigatranetexilat	UM	*CYP2C19*	15	0.2
	Edoxaban	PF	*ABCB1*	40	0.5
	Edoxaban	PF	*VKORC1*	29	0.3
	Edoxaban	PM	*CYP2C9*	7	0.1
	Edoxaban	PM	*CYP2C19*	3	0.0
	Edoxaban	UM	*CYP2C19*	12	0.1
	Phenprocoumon	PF	*ABCB1*	315	3.8
	Phenprocoumon	PF	*VKORC1*	215	2.6
	Phenprocoumon	PM	*CYP2C9*	47	0.6
	Phenprocoumon	PM	*CYP2C19*	30	0.4
	Phenprocoumon	UM	*CYP2C19*	63	0.8
	Prasugrel	PF	*ABCB1*	103	1.2
	Prasugrel	PF	*VKORC1*	89	11
	Prasugrel	PM	*CYP2C9*	17	0.2
	Prasugrel	PM	*CYP2C19*	8	0.1
	Prasugrel	UM	*CYP2C19*	29	0.3
	Rivaroxaban	PF	*ABCB1*	523	6.3
	Rivaroxaban	PF	*VKORC1*	369	4.4
	Rivaroxaban	PM	*CYP2C9*	97	1.2
	Rivaroxaban	PM	*CYP2C19*	70	0.8
	Rivaroxaban	UM	*CYP2C19*	109	1.3
	Ticagrelor	PF	*ABCB1*	148	1.8
	Ticagrelor	PF	*VKORC1*	79	1.0
	Ticagrelor	PM	*CYP2C9*	29	0.3
	Ticagrelor	PM	*CYP2C19*	17	0.2
	Ticagrelor	UM	*CYP2C19*	31	0.4
	Warfarin	PM	*CYP2C19*	1	0.0
CLD	Atorvastatin	PM	*CYP3A5*	827	43.2
	Atorvastatin	PF	*ABCB1*	200	10.4
	Atorvastatin	PF	*SLCO1B1*	22	1.1
	Fluvastatin	PM	*CYP3A5*	18	0.9
	Fluvastatin	PF	*ABCB1*	7	0.4
	Lovastatin	PM	*CYP3A5*	1	0.1
	Pravastatin	PM	*CYP3A5*	35	1.8
	Pravastatin	PF	*ABCB1*	8	0.4
	Pravastatin	PF	*SLCO1B1*	2	0.1
	Rosuvastatin	PM	*CYP3A5*	3	0.2
	Simvastatin	PM	*CYP3A5*	742	38.8
	Simvastatin	PF	*ABCB1*	194	10.1
	Simvastatin	PF	*SLCO1B1*	16	0.8
	Simvastatin	PM	*CYP3A4*	4	0.2

*A/T* anticoagulant/antiplatelet, *CLD* cholesterol-lowering drug, *ASS* acetylsalicylic acid, *PF* poor function, *PM* poor metabolizer, *UM* ultrarapid metabolizer.

## Discussion

For some cardiovascular drugs such as clopidogrel, warfarin, and simvastatin, robust data on clinical benefits due to preemptive pharmacogenetic testing provide a promising outlook for clinical implementation in routine care [3]. However, for guided therapy with most cardiovascular drugs, cost-effectiveness of pharmacogenetic testing in German routine care is not sufficiently elucidated yet. Therefore, the EMPAR study evaluates the genetic variability on the basis of participants’ metabolic profiles with regard to cardiovascular drugs and will consider pharmacoepidemiological evaluations concerning ADRs and pharmacoeconomic evaluations on the utilization and costs of health insurance services according to healthcare claims records in German routine care in future analyses. For this first evaluation, datasets of 10,748 participants were analyzed for basic population characteristics, DNA-quality control parameters and prevalence of pharmacogenetic variants which could be risk factors for ADRs.

In our study population, a tendency of an increased quantity of diagnoses and prescription entries in elderly participants compared to the young and moderate age participants was observed already at baseline (Supplement 3). This observation is concordant with studies evaluating age-specific trends such as comorbidity and polypharmacy [24–27]. A high percentage of detected prescriptions within the individual baseline year included inhibitors and antagonists of the cytochromes, the catalytic subunit, and transporters under study (Table 2). Especially here, phenotype prediction of the metabolizer status with regard to the affected medication of interest and the evaluation of ADRs could be compromised due to phenoconversion. Affected participants may have a higher risk of drug–drug, e.g., drug–drug–gene interactions concerning the medication of study interest [28, 29]. Furthermore, 21% of participants receiving ≥2 and especially 2.3% identified with ≥5 prescriptions in each quarter of the baseline year may be at higher risk of such interactions [30]. A previous study in the Netherlands concerning long-term polypharmacy (>240 days over a year) showed similar percentages of participants with long-term use of ≥2 drugs (20% elderly participants) and of more than 5 drugs (4% elderly participants) [31].

Therapeutic treatment with cardiovascular drugs in order to prevent coronary morbidity and mortality should also consider a risk reduction of ADRs [32]. Genetic risk factors can predict the individual response to particular drugs and the risk of ADRs to some extent. Around 95.5% of all participants are carrier of at least one relevant PGx variant (PharmGKB clinical annotation – Evidence level 1A, B). Thus, these data are comparable to other studies, for instance, reported by the eMERGE-PGx program where more than 96% of samples displayed relevant actionable PGx variants [33]. Furthermore, the Mayo RIGHT program showed that 99% of participants were carrier of at least one of such variants including also CYP2D6 variants and 3% of participants even were carrier of actionable PGx variants in *CYP2D6*, *CYP2C9*, *CYP2C19*, *SLCO1B1*, and *VKORC1* [3]. The analysis of genotype data of 11 genes of 44,000 participants of the Estonian biobank revealed that 99.8% of genotypes were associated with an increased risk profile with regard to at least one medication [34]. Thus, our study provided additional evidence that pharmacogenetic risk factors for ADRs are prevalent in a majority of representatives of an expected primarily Caucasian population sample.

Previous studies predicting metabolizer phenotypes in Caucasian populations reported that, e.g., in a Croatian population 3.98% of study participants were PMs for CYP2C9, while in a small Italian study population, 1.7% were PM due to homozygosity for the CYP2C19*2 variant [35, 36]. According to the PharmGKB CYP2C9 Frequency Table, a prevalence of 2.6% in a European population is estimated for a PM phenotype [37]. However, in the anticoagulant collective of the EMPAR study, a higher phenotype frequency was observed. Here 4% of participants presented with a CYP2C9 PM phenotype. Phenotype frequency information was not provided by PharmGKB for VKORC1 and is rarely reported in studies for Caucasian populations in Europe. In the EMPAR anticoagulant/antiplatelet collective 15.3% of study participants were predicted with a deficient VKORC1 phenotype due to homozygosity for the *VKORC1* rs9923231 TT variant. However, a lower frequency was reported by Schelleman et al. (Supplement 1 – Fig. 5) [38]. An evaluation of CYP2C19 predicted phenotypes concerning ethnic groups and the geographic regions across world populations showed that CYP2C19 PM phenotypes differed in Caucasian populations from 1.56% to 3.61%. Thereby, populations from Europe were represented with the lowest frequency of about 1.76% [39]. According to the PharmGKB CYP2C19 Frequency Table, CYP2C19 PMs are estimated in 2.3% of a European population [40]. Similar to these findings, PMs for CYP2C19 were detected in 2.6% of the EMPAR study population. The high concordance of our results with data from PharmGKB and data from literature concerning the distribution of phenotypes showed that the potential selection bias in this study due to many insurants that were excluded [16], mainly did not affect the phenotype frequencies predicted in this study.

CYP2C9 poor metabolism or VKORC1 poor enzymatic activity is associated with a higher risk of bleeding events upon treatment with several coumarins that were of primary study interest. Furthermore, in previous studies, CYP2C19 PMs were shown to be at higher risk of adverse cardiovascular events in treatment with clopidogrel [41]. In our study population, such phenotypes were present also in parallel with prescriptions of the respective cardiovascular drugs and could have increased the risk of ADRs for these participants.

Among drug–gene pairs relevant for the cholesterol-lowering drug cohort, for simvastatin-*SLCO1B1*, a risk of ADRs such as muscle problems including myalgias and rhabdomyolysis was reported in previous studies for the poor metabolism/function phenotype [42, 43]. However, relevant genes involved in statin transport or metabolism such as *SLCO1B1* (2.1.%) or *CYP3A4* (0.2%) occur in the cholesterol-lowering drug (statin) collective only as small fractions with that phenotype.

In future analyses, the impact of actionable variants, extreme phenotypes, and metabolic profiles on ADRs and cost expenditures in routine care will be investigated considering also low evidence level drug–gene pairs. Furthermore, concomitant medication and relevant associated pharmacogenes such as *CYP2D6* will be of additional interest concerning drug–drug–gene interactions. Screening for new relevant SNPs of pharmacogenes associated with ADRs will be performed especially considering the Y57.9!-diagnosis collective.

## Conclusion

Genetic analysis considering healthcare claims records provides valuable information to assess the risk of ADRs. Preemptive risk assessment probably gets even more important to elderly, comorbid patients under long-term polypharmaceutical therapies as identified in about 2.3% of the EMPAR study participants. On marker level, a majority of study participants were carrier of at least one actionable variant concerning pharmacogenes relevant for cardiovascular drugs. Furthermore, on metabolic profile level, important drug–gene combinations that could affect the safety of drug therapy were observed. With these and further evaluations, we aim to contribute to the accumulation of evidence regarding the clinical utility of pharmacogenetic markers to improve the information basis for reimbursement policy and adoption of preemptive testing into routine care in Germany.

## Supplementary information

Supplement 1

Supplement 2

Supplement 3
