# Supplementary material for: Evaluation of the EMPAR study population on the basis of metabolic phenotypes of selected pharmacogenes
Source: Pharmacogenomics J. 2022 Jan 31;22(2):136–44. doi: 10.1038/s41397-022-00268-6 (PMC8975744; doi:10.1038/s41397-022-00268-6)
Supplement: Supplementary file 1 — Supplement 1 [file 41397_2022_268_MOESM1_ESM.pdf]

Supplementary material

Table 1 Overview of investigated genes and genetic variants considered for the complete study population and the specific cardiovascular drug collectives. Genes marked in grey were only considered in quality control evaluations.

Collective	Gene	Marker	Collective	Gene	Marker
**Complete study population & Anticoagulant/antiplatelet collective**	*CYP2C19*	rs4244285	**Complete study population**	*CYP2D6*	rs72549353
	CYP2C19	rs28399504		*CYP2D6*	rs5030865
	CYP2C19	rs56337013		*CYP2D6*	rs5030656
	CYP2C19	rs4986893		*CYP2D6*	rs28371706
	CYP2C19	rs72552267		*CYP2D6*	rs72549354
	CYP2C19	rs41291556		*CYP2D6*	rs1135840
	CYP2C19	rs72558186		*CYP2D6*	rs5030862
	CYP2C19	rs12248560		*CYP2D6*	rs16947
	CYP2C9	rs7900194		*CYP2D6*	rs5030867
	CYP2C9	rs72558187		*CYP2D6*	rs1065852
	CYP2C9	rs1057910		*CYP2D6*	rs35742686
	CYP2C9	rs9332239		*CYP2D6*	rs28371725
	CYP2C9	rs9332131		*CYP2D6*	rs5030655
	CYP2C9	rs72558190		*CYP2D6*	rs3892097
	CYP2C9	rs28371685		*CYP2D6*	rs59421388
	CYP2C9	rs56165452		*CYP2D6*	rs28371735
	CYP2C9	rs28371686		*CYP2D6*	rs5030863
	CYP2C9	rs72558188		*CYP2D6*	rs72549357
	CYP2C9	rs1799853		*CYP2D6*	dup4125_4133
	VKORC1	rs9923231		*CYP1A2*	rs762551
**Complete study population & Anticoagulant/antiplatelet collective & Cholesterol lowering drug collective**	*ABCB1*	rs1045642		*CYP1A2*	rs56107638
**Complete study population & Cholesterol lowering drug**	*SLCO1B1*	rs4149056		*CYP1A2*	rs12720461
	CYP3A4	rs55785340		*CYP1A2*	rs2069514
	CYP3A4	rs35599367		*CYP1A2*	rs72547513
	CYP3A4	rs4987161		*CYP2B6*	rs28399499
	CYP3A5	rs776746		*CYP2B6*	rs3745274
	CYP3A5	rs28365083			
	CYP3A5	rs41303343			
	CYP3A5	rs10264272			

Quality control plots of PGx Data (Agena Bioscience)

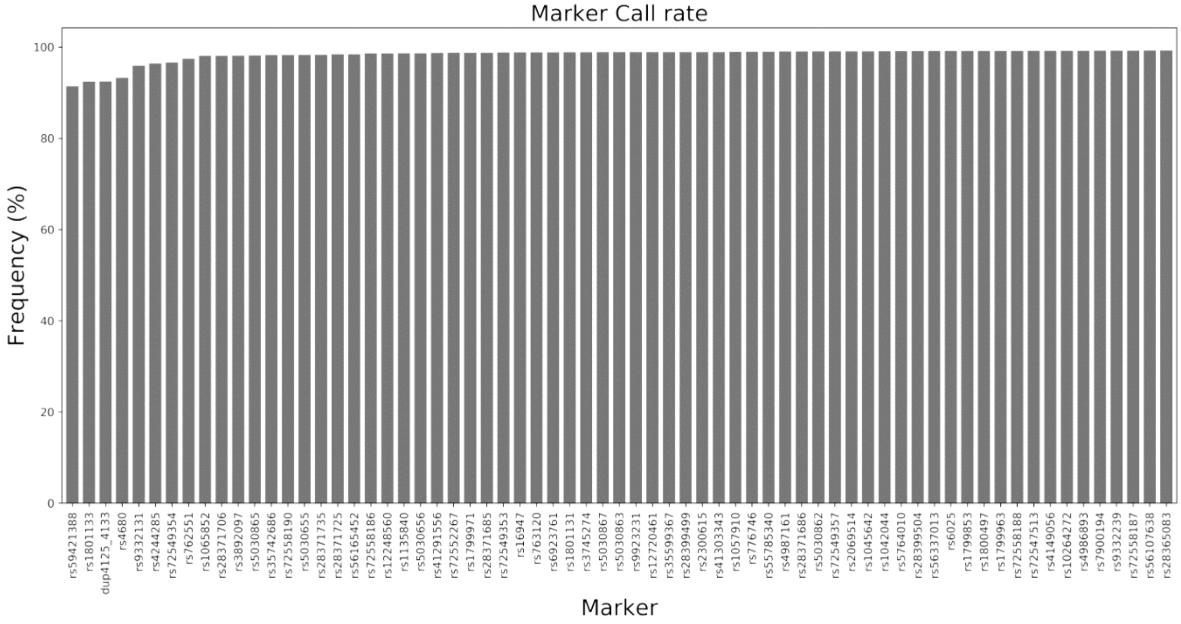

Figure 1 Marker call rates of all investigated markers (Agena Bioscience)

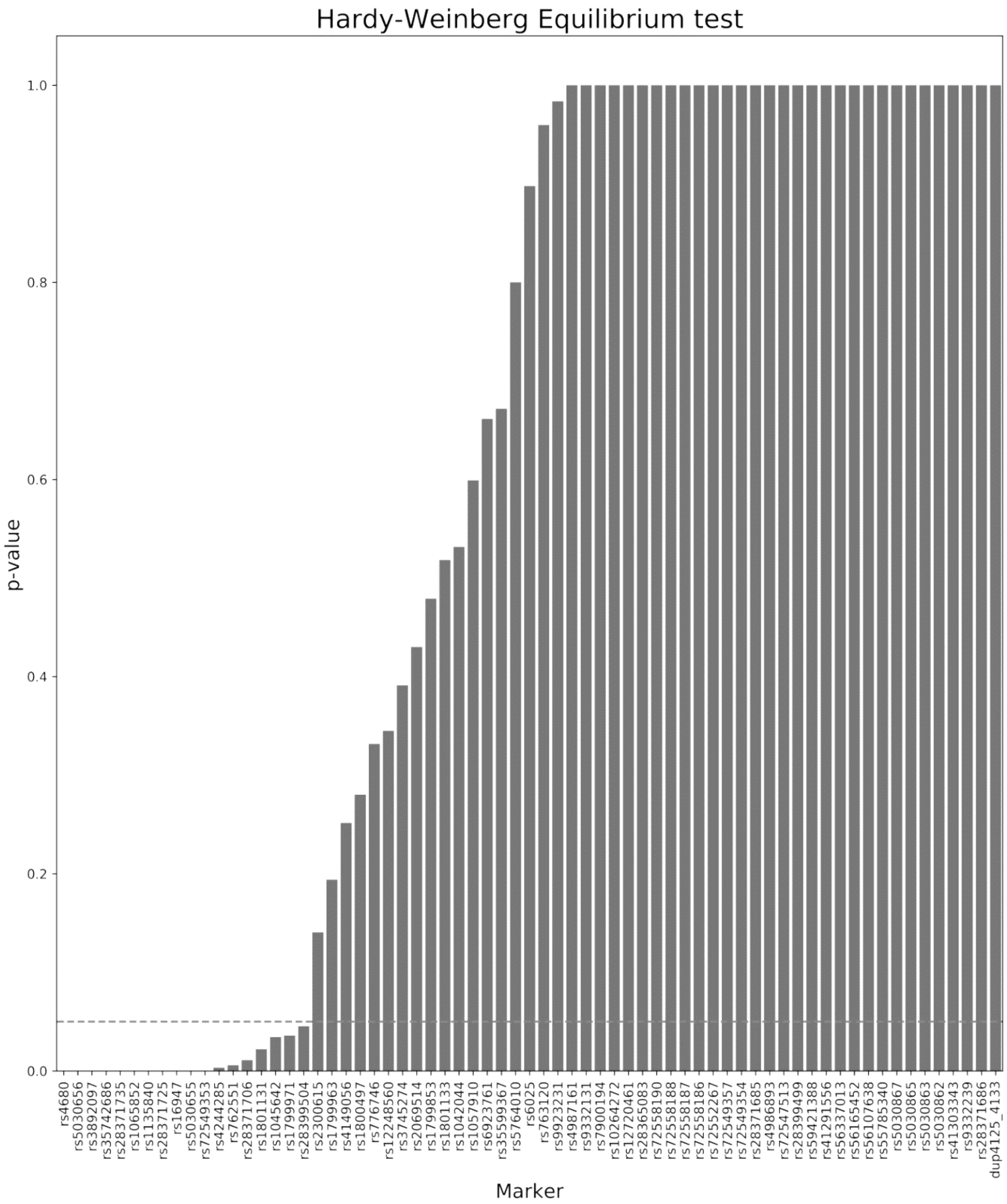

Figure 2 Hardy Weinberg Equilibrium test results of all investigated markers. For *CYP2D6* 10 of 17 markers were observed to be not in equilibrium. Several variants of that fraction represent reduced function or non-functional alleles. Especially the markers rs5030656 (*9) and rs5030655 (*6) represent specific variants and were observed with low frequencies among Europeans (Whirl-Carrillo et al, 2012). Furthermore, two markers were observed for *CYP2C19* which are not in equilibrium as well. Among them, rs4244285 (*2) encoding for poor functional- and rs28399504 (*4) encoding for non-function activity. In addition, rs762551 (*CYP1A2*) was not accounted to be in HWE.

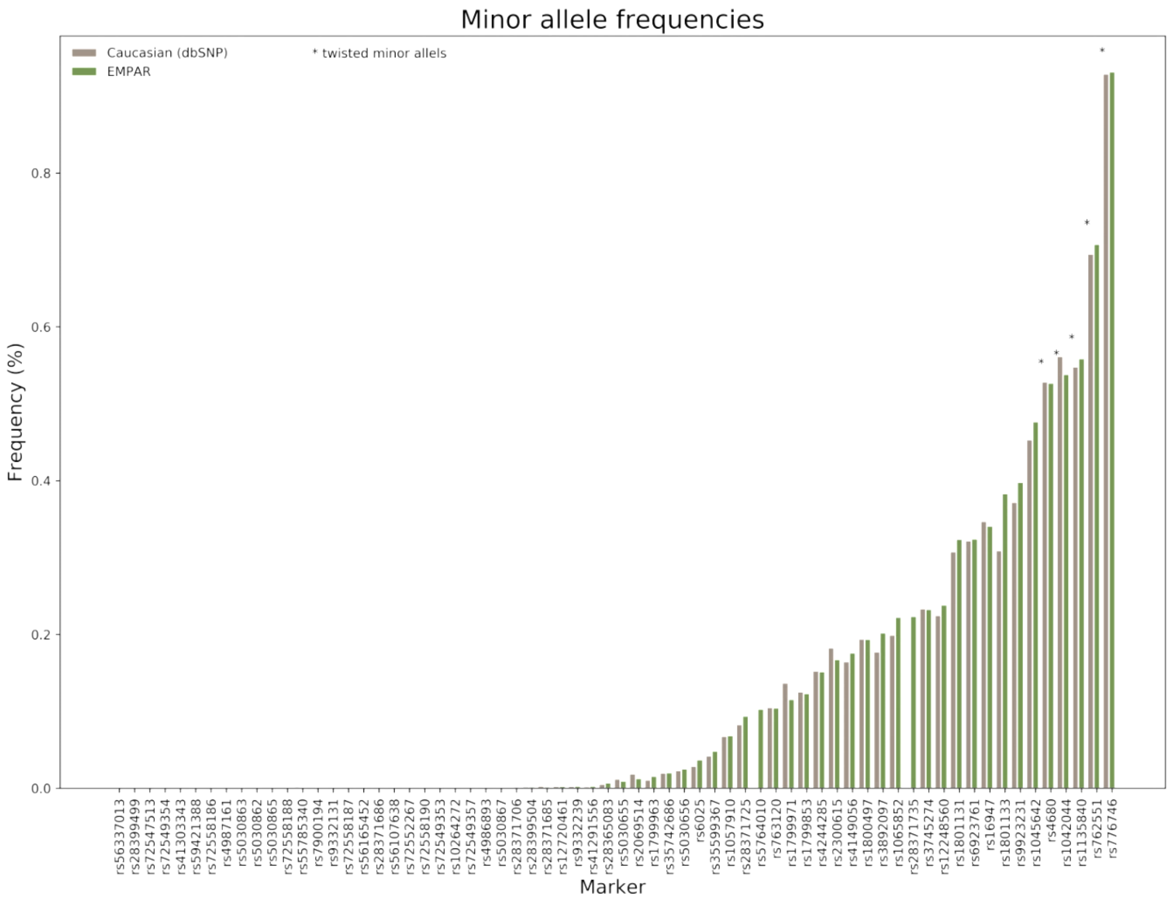

Figure 3 Minor allele frequencies of all investigated markers compared to Caucasian dbSNP frequencies

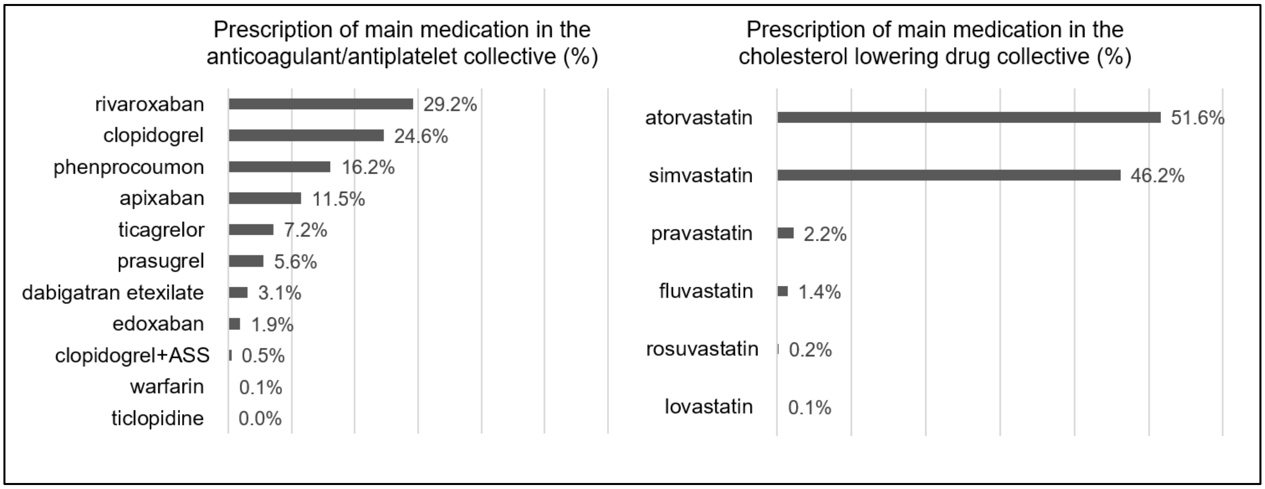

Figure 4 Percentage of the most frequent prescriptions of main medication in the anticoagulant/antiplatelet and the cholesterol lowering drug collective. ASS: acetylsalicylic acid.

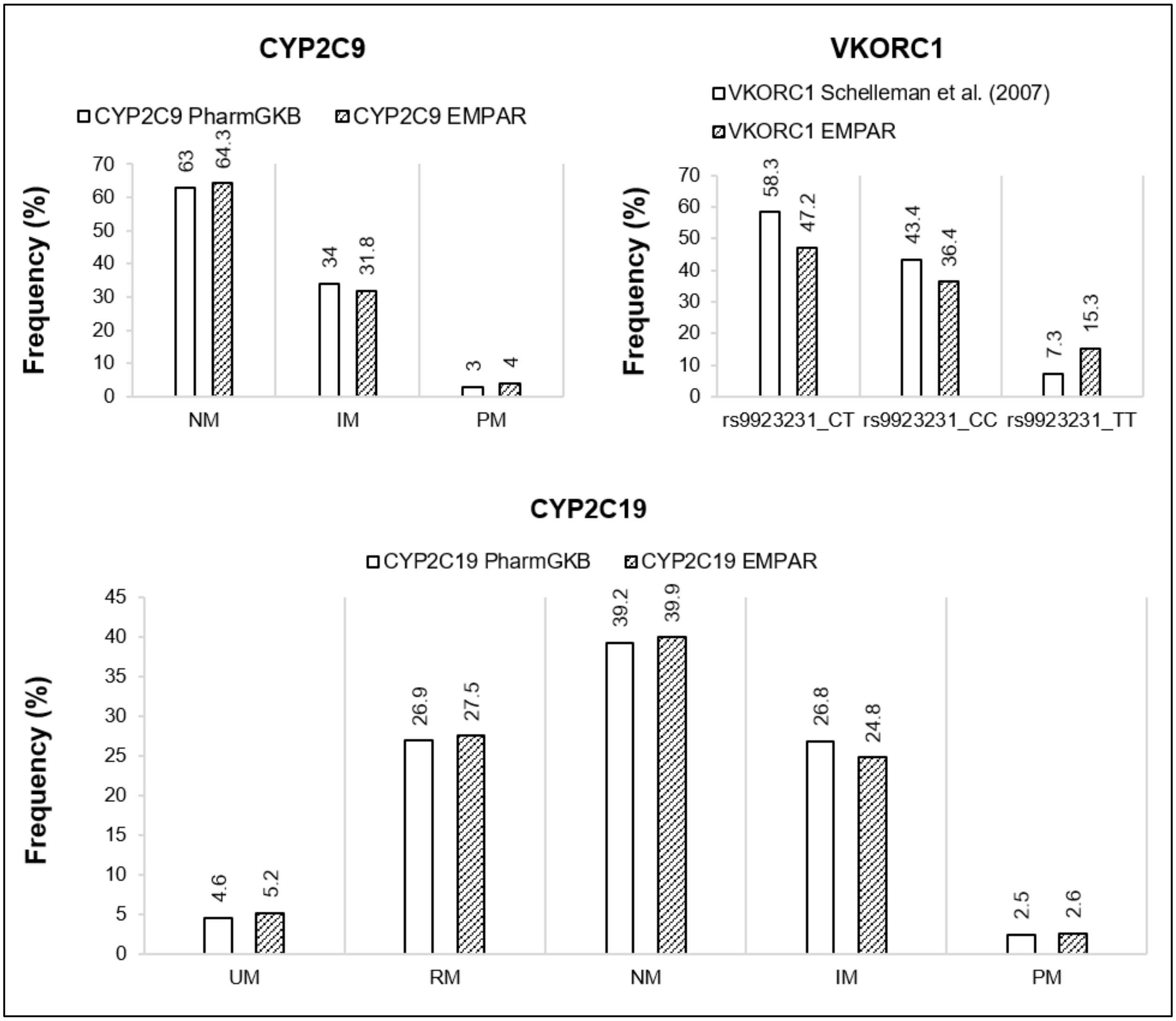

Figure 5 Frequencies of computed metabolic profiles of genes involved in anticoagulant/antiplatelet metabolism (*CYP2C19, CYP2C9* and *VKORC1*) compared to reference data. UM: ultra-rapid metabolizer, RM: rapid metabolizer, NM: normal metabolizer, IM: intermediate metabolizer, PM: poor metabolizer

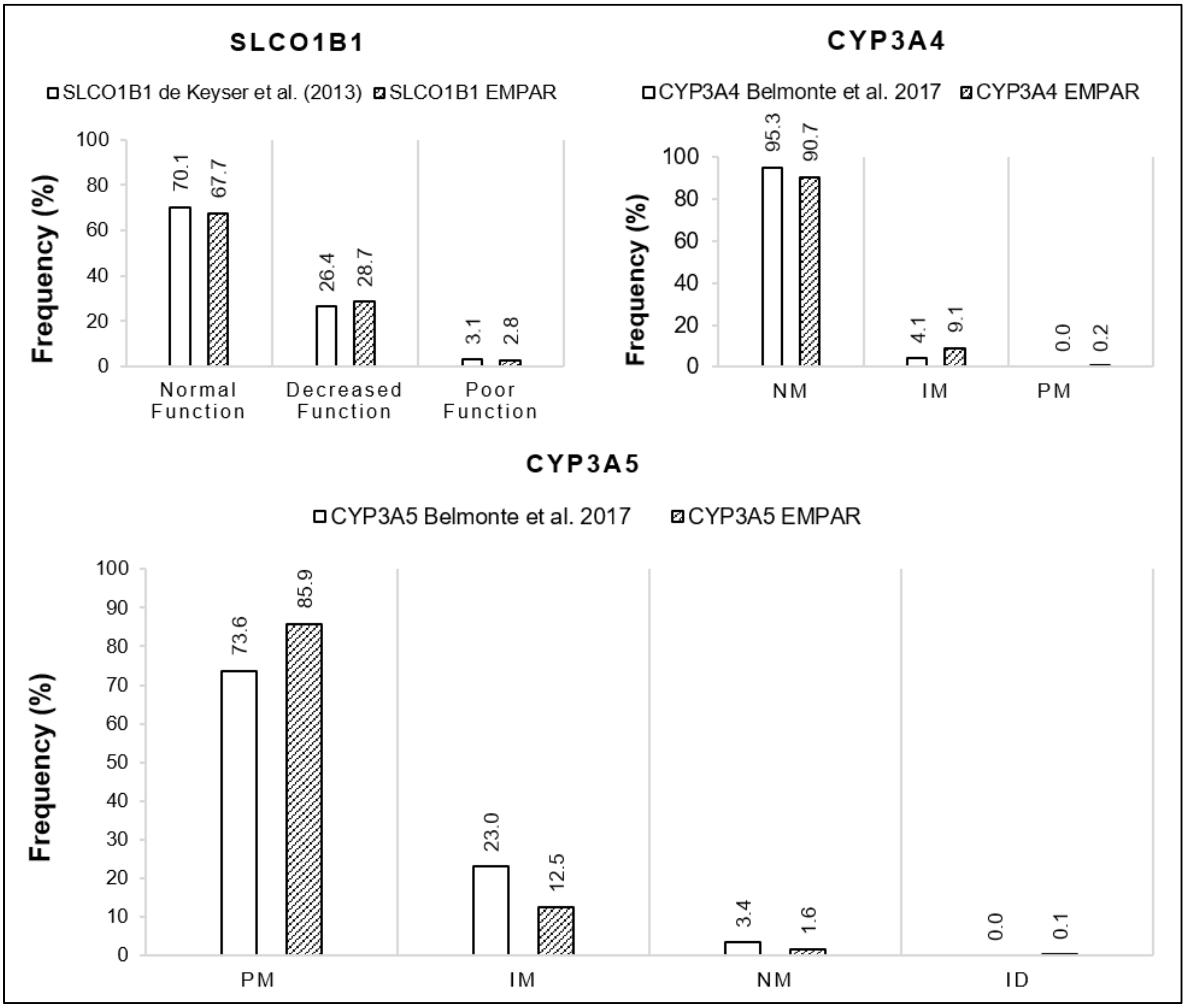

Figure 6 Frequencies of computed metabolic profiles involved in statin metabolism or transport (*CYP3A4, CYP3A5* and *SLCO1B1*) compared to reference data. NM: normal metabolizer, IM: intermediate metabolizer, PM: poor metabolizer, ID: intermediate metabolizer

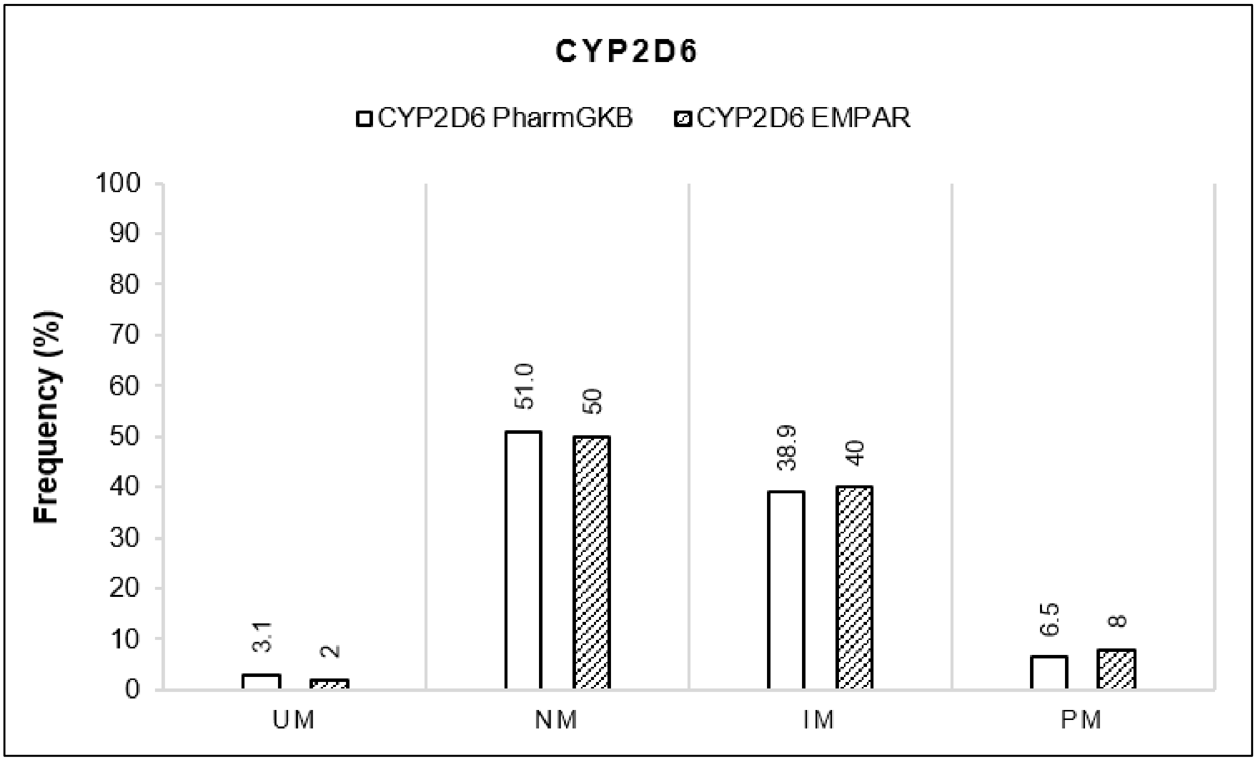

Figure 7 Frequencies of computed metabolic profiles (*CYP2D6*) compared to reference data. UM: ultra-rapid metabolizer, NM: normal metabolizer, IM: intermediate metabolizer, PM: poor metabolizer. The VeriDose CYP2D6 CNV panel was applied to compute the ultra-rapid metabolizer status (UM) for *CYP2D6*. In addition, this panel reports the (*CYP2D6-CYP2D7*) hybrid allele information for Exon9, *68 and *13 variants. The overall phenotype computation was improved by this information.

Diplotype-Diplotype combinations per collective

Anticoagulant/antiplatelet collective

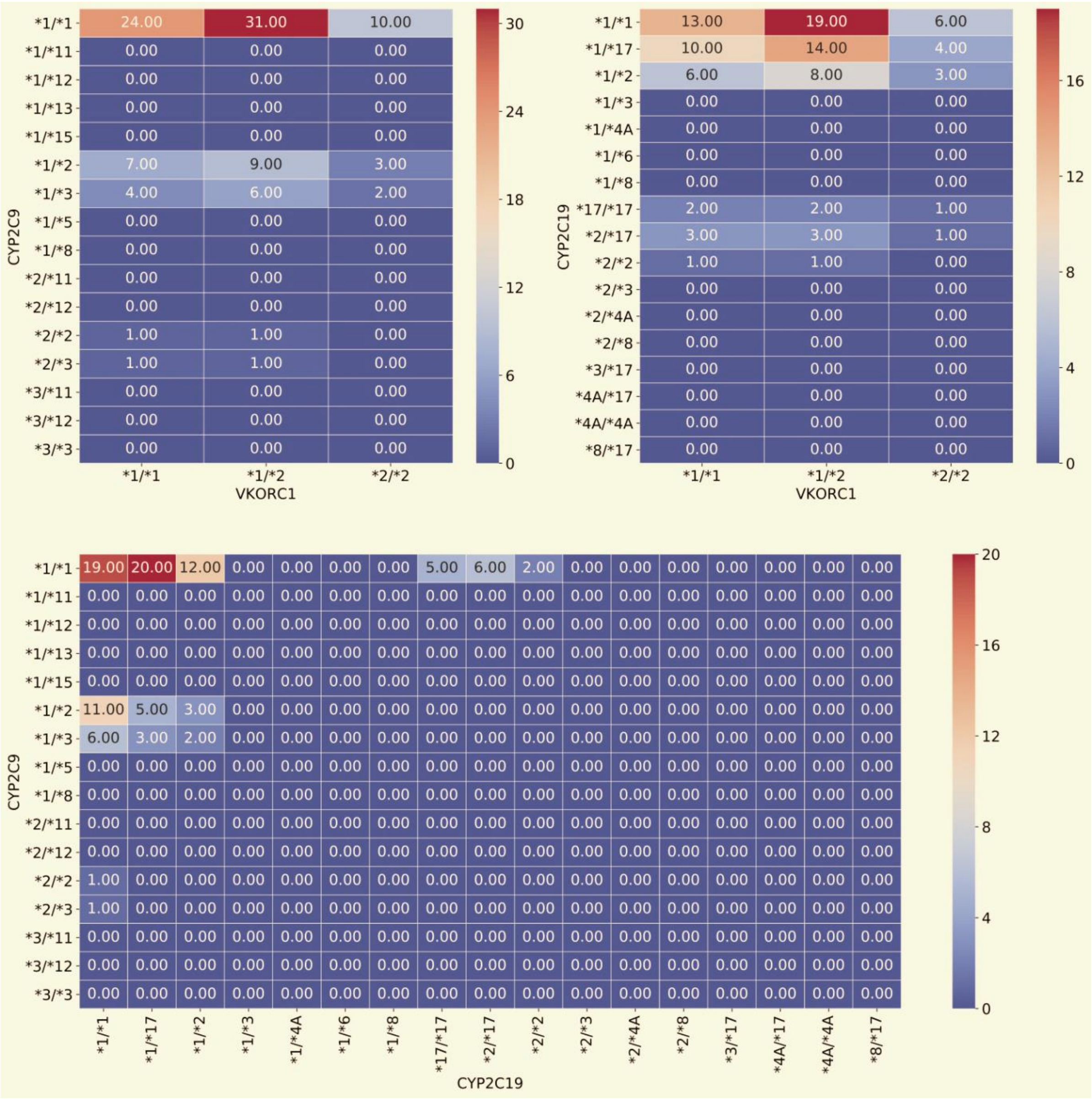

Figure 8 Frequencies (%) of Diplotype-Diplotype combinations of *CYP2C9*, *CYP2C19* and *VKORC1*. Highest frequencies are represented by *CYP2C9**1/*1 with *CYP2C19**1/*17 and *CYP2C9**1/*1 with *CYP2C19**1/*. For *VKORC1*, the most frequent combination was *1/*1 and *1/*2 (*CYP2C(1)9* and *VKORC1*). The highest haplotype deviations from the normal function were *2 for *CYP2C9*/*VKORC1* (decreased function) and *17 (increased function) for *CYP2C19* with potential effect on drug therapy.

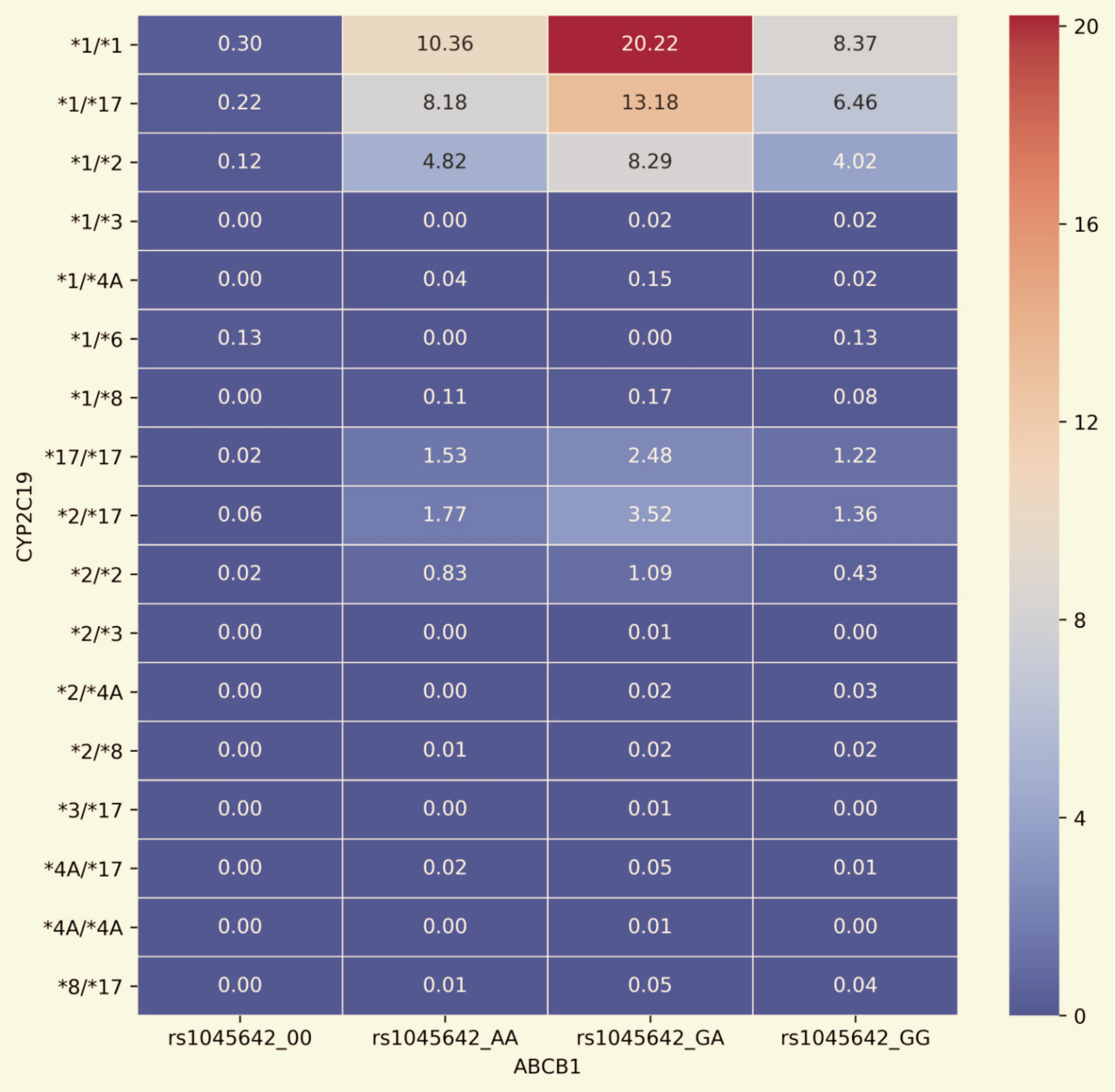

Figure 9 Frequencies (%) of Diplotype-Diplotype combinations between *ABCB1* and *CYP2C19*

Cholesterol lowering drug collective

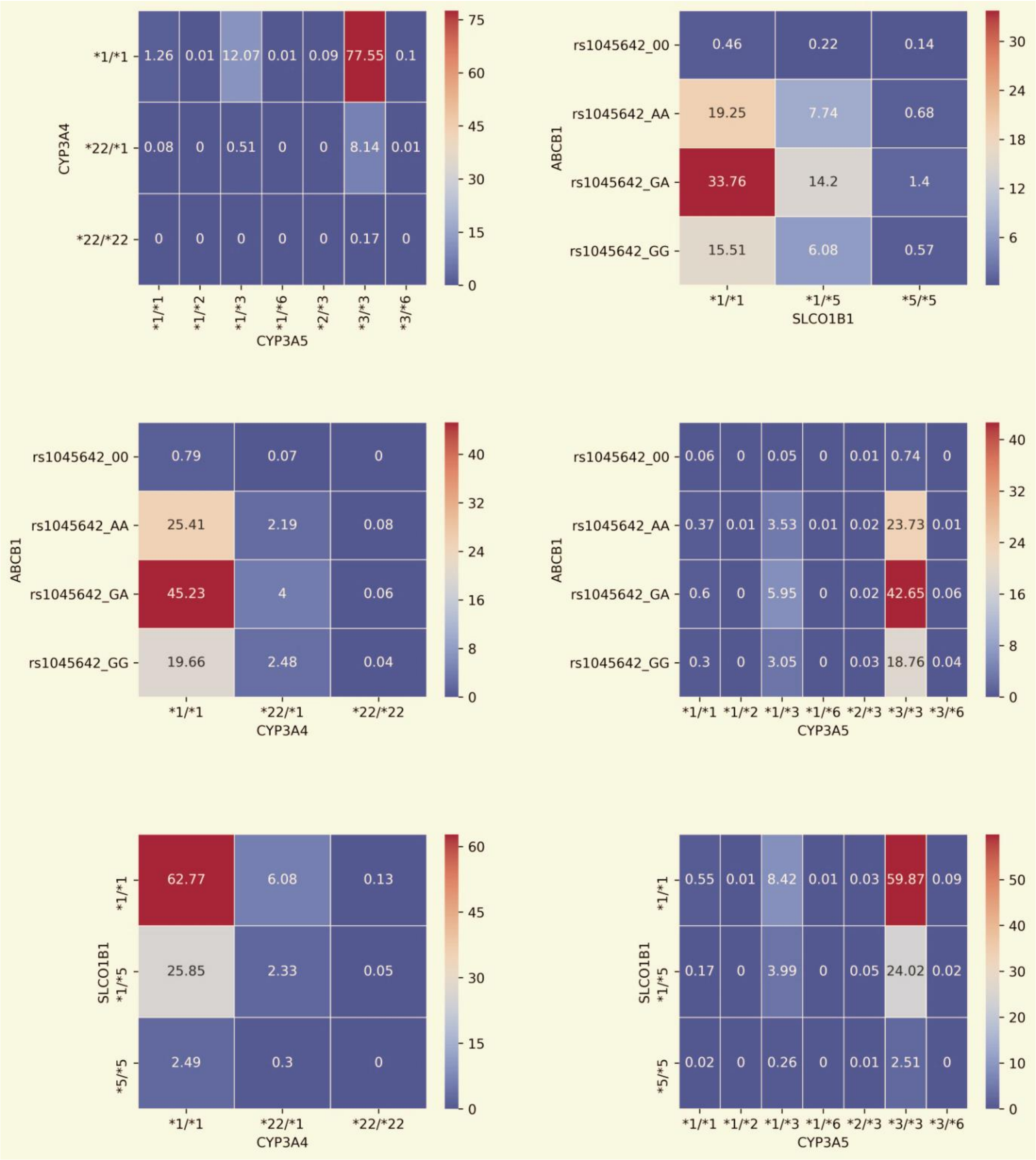

Figure 10 Frequencies (%) of Diplotype-Diplotype combinations between *ABCB1*, *CYP3A4*, *CYP3A5* and *SLCO1B1*. Highest frequencies could be observed in combination with the *CYP3A5* *3/*3 (poor function) diplotype. In the EMPAR study *CYP3A5* *3/*3 with *CYP3A4* *1/*1 (normal function) were the most frequent observed combination. Similar results were obtained for *SLCO1B1* *1/*1 to any other gene combination analyzed, whereas a combination with the *ABCB1* transporter rs1045642_GA (MW) diplotype appeared as most prevalent result.

Note:

Data from de Keyser et al. (2013)[1], Ufer et al. (2009)[2] and Belmonte et al. (2017)[3] were analogous to EMPAR data translated into phenotypes.

1. de Keyser CE, Peters BJM, Becker ML, et al. The SLCO1B1 c.521T>C polymorphism is associated with dose decrease or switching during statin therapy in the Rotterdam Study. Pharmacogenet Genomics. 2014;24(1):43-51. doi:10.1097/FPC.0000000000000018

2. Ufer M, Mosyagin I, Muhle H, et al. Non-response to antiepileptic pharmacotherapy is associated with the ABCC2-24C>T polymorphism in young and adult patients with epilepsy. Pharmacogenet Genomics. 2009;19(5):353-362. doi:10.1097/FPC.0b013e328329940b

3. Belmonte C, Ochoa D, Román M, et al. Influence of CYP2D6, CYP3A4, CYP3A5 and ABCB1 Polymorphisms on Pharmacokinetics and Safety of Aripiprazole in Healthy Volunteers. Basic Clin Pharmacol Toxicol. 2018;122(6):596-605. doi:10.1111/bcpt.12960
